# Amoxicillin vs. ceftriaxone to treat pneumonia caused by amoxicillin-non-susceptible *Streptococcus pneumoniae*

**DOI:** 10.1128/aac.00780-25

**Published:** 2025-08-13

**Authors:** Daniel M. Musher

**Affiliations:** 1 Department of Medicine, Baylor College of Medicine, Houston, Texas, USA; 2 Department of Microbiology and Molecular Virology, Baylor College of Medicine, Houston, Texas, USA; 3 Medical Care Line (Infectious Disease Section), Michael E. DeBakey Veterans Affairs Medical Center, Houston, Texas, USA; Houston Methodist Hospital and Weill Cornell Medical College, Houston, Texas, USA

**Keywords:** pneumonia, *Streptococcus pneumoniae*, amoxicillin, ceftriaxone

## Abstract

Although the article by J Càmara, I Grau, A González-Díaz, N Santos, et al. (Antimicrob Agents Chemother 69:e00237-25, 2025, https://doi.org/10.1128/aac.00237-25) suggests that ceftriaxone is greatly superior to amoxicillin in treating pneumonia due to pneumococci with an MIC > 2 mg/L, much of the difference in efficacy of the two drugs may have been due to dosage, route, and times of administration of the amoxicillin.

## COMMENTARY

Although sulfapyridine was the first antimicrobial agent that successfully treated pneumococcal pneumonia (1), the miraculous antibiotic era really began in 1941 when Abraham et al. (2) treated a series of patients with benzylpenicillin G to cure infections caused by *Staphylococcus aureus*. Very soon thereafter, penicillin was shown to be enormously successful in treating pneumonia and other infections caused by *Streptococcus pneumoniae* (3). There even was some degree of success when penicillin was given orally (4), although drug absorption from the gut was known to be unreliable.

The introduction of amoxicillin in 1972 opened a new chapter in the use of beta-lactam antibiotics for pneumococcal infections. Unlike penicillin or ampicillin, this drug was reliably absorbed after oral administration and, thanks to its half-life of nearly 1 hour and an MIC of 0.02 µg/mL for pneumococci (5), 500 mg administered every 6–8 hours could be counted on to treat nearly all non-meningeal infections caused by *S. pneumoniae*. Unfortunately, within the next few years, pneumococcal strains with decreased susceptibility to penicillin began to be identified around the world, culminating in the appearance, in South Africa in 1976, of pneumococci for which the MBC of penicillin ranged from 2 to 10 µg/mL, depending upon the inoculum size utilized for *in vitro* studies (6). This level of resistance *in vitro* translated to treatment failure *in vivo*.

Serotypes that were most likely to be resistant were those prevalent as carriage strains and as causes of pneumococcal infection in children. The therapeutic problem of pneumococcal resistance was greatly ameliorated after 2000 by the widespread adoption—in infants and young children—of a seven-valent conjugate pneumococcal vaccine (7), which not only stimulated humoral immunity, thereby protecting recipients against infection, but also stimulated mucosal immunity, greatly reducing carriage of strains with reduced penicillin susceptibility. Reduced carriage in children decreased the spread of these relatively resistant pneumococci to adults, an indirect or “herd” effect of vaccination (8).

**Fig 1:**
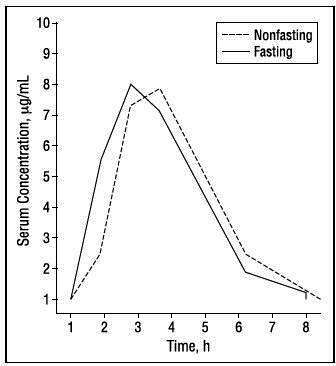
Mean serum concentrations of amoxicillin in 12 healthy young men after an oral dose of 500 mg (11).

In their comprehensive study, “Amoxicillin-non-susceptible *S. pneumoniae* causing invasive pneumoniae: serotypes, clones, and clinical impact,” Càmara et al. (9) report that the proportion of pneumococci that are not susceptible to 2 µg/mL amoxicillin at the Bellvitge Hospital in Barcelona, Spain, has decreased from 27.1% before 2001 to 8.6% in 2016–2020. These authors point out the major discrepancy between definitions of resistance to amoxicillin using CLSI and European Committee on Antimicrobial Susceptibility Testing (EUCAST) criteria and select a definition of resistance as MIC >2 mg/L, one that is similar to our recommendation (10) and fits much better with achievable drug levels following oral administration than either CLSI or EUCAST recommendations (Fig. 1).

A number of points that are important for clinicians can be made based on this approach. Using MIC >2 mg/L as a definition of resistance, the mortality in patients who received amoxicillin for invasive disease due to amoxicillin-resistant pneumococcus was six times greater than in those who were treated with a third-generation cephalosporin. Importantly, Camara et al. did not address dosage, dosing intervals, and route of administration of amoxicillin (except to state that some of their patients received amoxicillin orally), all of which can have a major impact on drug levels and, therefore, on the effectiveness of therapy, especially at higher pneumococcal MICs.

As can be seen in Fig. 1 and confirmed by de Velde et al. (12), administering amoxicillin orally every 12 hours could readily be predicted to fail in some proportion of healthy young adults. Carlier et al. (13) documented even lower blood levels after administering amoxicillin to critically ill patients. For these reasons, I do not use amoxicillin 875 mg every 12 hours to treat any pneumococcal infection, despite its being regarded as acceptable. A drug with a half-life of 1 hour should only be given every 12 hours to treat the most exquisitely susceptible of organisms. It is important to underscore the obvious point that means are determined from a range of values, so some proportion of patients always achieve drug levels below the reported mean.

In contrast, ceftriaxone is only given intravenously and has a half-life of 6 hours. A 1 gm dose given to an average-size adult once every 24 hours maintains levels well above 2 mg/L for the entire treatment period (resistance of *S. pneumoniae* to this drug is defined as MIC >2 mg/L). This observation alone should lead to the conclusion reached by Camara et al., namely that the use of ceftriaxone to treat pneumonia caused by a relatively amoxicillin-resistant pneumococci with this drug might well lead to better outcomes.

Current guidelines from the American Thoracic Society and the Infectious Diseases Society of America (14) recommend 1.5 g ampicillin with sulbactam intravenously every 6 hours for initial empiric treatment of community-acquired pneumonia in hospitalized patients. Amoxicillin is not given intravenously in the US. This drug and ampicillin are regarded as equivalent, and American guidelines recommend ampicillin. The importance of giving the initial antibiotic dose intravenously to patients hospitalized for pneumonia is to avoid the delay inherent in reaching appropriate tissue levels when treatment is given orally, and the possibility that a patient sick enough to be hospitalized may slide into shock before an orally administered drug is sufficiently absorbed. The recommended dose will maintain effective antibiotic levels in the bloodstream for 60% of the day against all but the most highly resistant pneumococci. De-escalation to 500 mg or 1 g orally every 6 hours should then successfully complete therapy.

In conclusion, although the retrospective study by Càmara et al. (9) suggests that amoxicillin may be inferior to ceftriaxone in treating pneumococcal pneumonia, it is likely that, in that real-world setting, insufficient dosing was responsible for some proportion of the poor results with amoxicillin. When given in doses recommended by US guidelines, ampicillin or amoxicillin is expected to be as effective as ceftriaxone in treating the great majority of cases of pneumococcal pneumonia.
